# Experimental Evaluation of Sub-Sampling IQ Detection for Low-Level RF Control in Particle Accelerator Systems

**DOI:** 10.3390/s22010038

**Published:** 2021-12-22

**Authors:** Tomasz Kowalski, Gian Piero Gibiino, Jarosław Szewiński, Krzysztof Czuba, Dominik Rybka, Konrad Chmielewski, Zbigniew Wojciechowski, Maciej Sitek

**Affiliations:** 1 National Centre for Nuclear Research (NCBJ), 05-400 Otwock, Poland; J.Szewinski@ncbj.gov.pl (J.S.); Dominik.Rybka@ncbj.gov.pl (D.R.); Konrad.Chmielewski@ncbj.gov.pl (K.C.); Zbigniew.Wojciechowski@ncbj.gov.pl (Z.W.); Maciej.Sitek@ncbj.gov.pl (M.S.); 2 Institute of Electronic Systems, Warsaw University of Technology, 00-661 Warsaw, Poland; Krzysztof.Czuba@pw.edu.pl; 3 Department of Electrical, Electronic, and Information Engineering “Guglielmo Marconi”, University of Bologna, 40136 Bologna, Italy; gianpiero.gibiino@unibo.it

**Keywords:** low-level rf, linear accelerator, phase noise

## Abstract

The low-level radio frequency (LLRF) control system is one of the fundamental parts of a particle accelerator, ensuring the stability of the electro-magnetic (EM) field inside the resonant cavities. It leverages on the precise measurement of the field by in-phase/quadrature (IQ) detection of an RF probe signal from the cavities, usually performed using analogue downconversion. This approach requires a local oscillator (LO) and is subject to hardware non-idealities like mixer nonlinearity and long-term temperature drifts. In this work, we experimentally evaluate IQ detection by direct sampling for the LLRF system of the Polish free electron laser (PolFEL) now under development at the National Centre for Nuclear Research (NCBJ) in Poland. We study the impact of the sampling scheme and of the clock phase noise for a 1.3-GHz input sub-sampled by a 400-MSa/s analogue-to-digital converter (ADC), estimating amplitude and phase stability below 0.01% and nearly 0.01°, respectively. The results are in line with state-of-the-art implementations, and demonstrate the feasibility of direct sampling for GHz-range LLRF systems.

## 1. Introduction

Free electron lasers, like the Polish Free Electron Laser (PolFEL) [1] now under development at the National Centre for Nuclear Research (NCBJ) in Poland, are an advanced tool for a range of scientific, industrial, and medical applications [2], allowing researchers to examine materials, characterize biological samples, etc. Such lasers are based on highly concentrated electron beams accelerated in resonant cavities, used to generate high-energy coherent light at wavelengths and peak powers that are difficult to achieve with conventional methods, e.g., the ones based on optical resonators [3,4].

High-precision radio-frequency (RF) electronics are widely used in linear accelerators [5] to ensure the stability of the accelerating electromagnetic (EM) field. The requirements for the amplitude and phase stability of the field can range from around 1% to 0.1% in amplitude and from 1° to 0.1° in phase [6,7,8], or even up to 0.01% and 0.01°, respectively, in the most demanding cases [9]. These requirements are derived from the desired beam parameters of the accelerator, e.g., bunch-to-bunch energy spread [10], and must be satisfied for an observation time corresponding to the RF pulse width in which electron bunches are accelerated, typically in the order of tens of milliseconds (in the case of PolFEL, the exact requirements are not yet defined at the time of writing, as the beam dynamics are still being calculated).

A fundamental component of the accelerator is the low-level radio frequency (LLRF) system, which is a feedback controller leveraging on precise measurements of the EM field. A key part of the LLRF system is the EM field detection inside the resonant cavity, which is carried out using In-phase/Quadrature (IQ) demodulation of the RF signals yielding the amplitude and phase of the accelerating field.

Traditionally, IQ detection is performed using an analogue frequency downconverting stage (i.e., a mixer) and an analogue-to-digital converter (ADC). However, direct sampling of RF signals has recently become feasible for the GHz range by using modern high-performance broadband ADCs [11,12]. In principle, direct sampling allows for a straightforward low-latency solution eliminating the need for analogue downconversion, which is a typical source of instability (due to, e.g., long-term temperature drift) often requiring additional calibrations [13].

The goal of this work is to experimentally evaluate direct sampling approaches utilizing a high-bandwidth, fast-sampling ADC for the purpose of the LLRF system of the PolFEL, operating at the resonant frequency of 1.3 GHz. In particular, we aim at comparing different sampling frequencies as well as at examining the impact of clock signal phase noise on the acquisition in order to estimate the best achievable amplitude and phase stability for the system under development.

This article is structured as follows. Section 2 describes the working principles of an LLRF system and presents its general structure and components. In Section 3, suitable RF signal acquisition methods are discussed by presenting different IQ detection and demodulation schemes. Section 4 shows the prototype LLRF system under development for the PolFEL, which is here used in the evaluation of the RF signal detection. In Section 5, a metrological characterization of the utilized ADC-based receiver is carried out. Finally, Section 6 presents the evaluation of amplitude and phase stability of the receiver. The influence of clock signal phase noise is examined by comparing the performance of different sampling schemes. Conclusions are drawn in Section 7.

## 2. Low-Level RF System

Particle accelerators utilize EM field for accelerating charged particles (e.g., electrons) in narrowband (e.g., 300 Hz bandwidth) resonant cavities with fundamental frequencies ranging from tens of MHz to several GHz, depending on the type of cavity and the final application [14,15,16,17]. The electric component of the EM field with given amplitude and phase interacts inside the cavity with the charged particles which, in turn, get accelerated (or decelerated, depending on the phase). Indeed, a precise control of amplitude and phase of the EM field inside the cavity is of primary importance to maximize acceleration. The accurate control of the EM field involves the calibration of its amplitude and phase as well as ensuring field stability throughout the acceleration process. Several external factors including charge fluctuations, microphonics, or de-tuning due to the Lorenz force [18,19] can disturb the field during operation.

**Figure 1:**
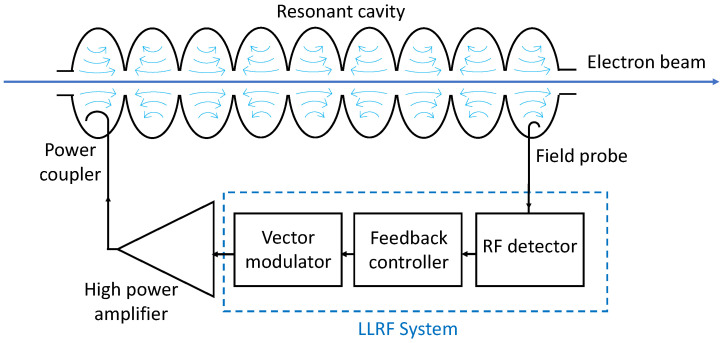
Block diagram of a single-cavity (9-cell) LLRF system.

These sources of non-ideality can be tackled by the LLRF system, which implements a closed-loop control feedback aimed at the stabilization of the field parameters in the cavity. The fundamental elements of a single-cavity LLRF system, whose block diagram is reported in Figure 1, are an RF field detector, a digital feedback controller, and an RF actuator. A pick-up probe is utilized to couple a small portion of the field power inside the cavity to the input of the LLRF system. The RF detector is an acquisition circuit implementing IQ demodulation of the RF field in order to retrieve the relevant values of amplitude and phase. Several detection schemes are suitable for this purpose, as will be discussed later in the article (see Section 3).

The LLRF controller, which is implemented as a digital circuit, is typically realized in field-programmable gate array (FPGA) for low-latency operation, high performance, and flexibility. In the commonly used generator-driven resonator (GDR) control mode, the measured values are compared with pre-calculated set-point values, and the resulting deviations are used to adjust output amplitude and phase. This function may be implemented in the form of a typical feedback controller, such as a proportional-integral (PI) controller or a proportional-integral-derivative (PID) controller [20,21]. Depending on the architecture for the signal acquisition, the controller can operate on the amplitude and phase calculated from the demodulated signals, or directly on the IQ signals to minimize the processing latency. The lower latency is particularly advantageous, as it allows for higher feedback gain and more flexibility in control algorithms, e.g., for additional filtering stages.

The output of the controller is then fed to a signal actuator, which is used to modulate a low-phase-noise reference RF signal. The actuator is most commonly an IQ modulator driven by two digital-to-analogue converters (DACs) for the I and Q signals, respectively. However, direct digital synthesis (DDS) of the digitally modulated signal is also possible, and it has been used in some lower frequency systems, e.g., in the 80–200 MHz range [22,23].

The output of the actuator drives a high power RF amplifier. For high accelerating gradients, the RF amplifier technology commonly in use is a Klystron with RF output powers in the order of several MW, suitable for driving multiple cavities at once. In such a configuration, vector sum control [18] is necessary to reconstruct the LLRF input signal from multiple cavities. Conversely, solid-state power amplifiers (SSPA) or inductive output tubes (IOT) can be used for lower power systems in the kW range. Typically, SSPAs and IOTs are used for driving individual cavities [24] in single-cavity regulation mode, as it is the case for the accelerator studied in this work. Finally, the amplified and modulated RF signal at the output of the LLRF system is fed to the cavity via a power coupler.

## 3. RF Signal Detection Methods

In order for the LLRF system to guarantee phase and amplitude stability, the RF detector must provide sufficient accuracy in sensing field disturbances or long-term drifts occurring within the cavity. The fidelity of the acquisition process is then critical for the overall performance of an LLRF system [12]. In the following, we introduce the main IQ detection schemes to be possibly used in the accelerator system.

### 3.1. IQ Demodulation Schemes

**Figure 2:**
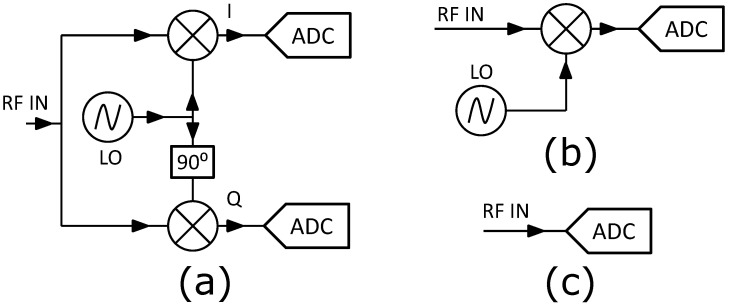
Block diagrams of typical IQ detection schemes: (**a**) analogue IQ demodulation via a quadrature demodulator; (**b**) digital IQ demodulation with analogue downconversion; (**c**) digital IQ demodulation with direct sampling.

Traditionally, IQ demodulation was carried out by means of an analogue IQ demodulator [25,26], where the IQ components are obtained by mixing the input signal with a local oscillator (LO) signal and with the same LO signal shifted by 90°, respectively, (see Figure 2a).

The resulting I and Q signals are then sampled by two separate ADCs. In this basic configuration, several technological parameters can impact the measurement accuracy, e.g., gain and offset variability across the two mixers, phase imbalance in power splitters, phase noise of the local oscillator, and distortion due to the receivers [27].

Alternatively, IQ detection can be carried out by analogue frequency downconversion combined with digital demodulation, as shown in Figure 2b. In this case, the input signal is downconverted to an Intermediate Frequency (IF) and sampled using a single ADC receiver [9,24]. Then, the acquired data is digitally post-processed to eventually extract the I and Q samples. The advantages of this method consist of the removal of any technological deviations across the two downconverting paths of Figure 2a, as well as the use of just one ADC-based receiver. Nevertheless, the non-linearity of the mixer as well as its long-term drifts (due to, e.g., temperature) still remain as sources of non-ideality.

**Figure 3:**
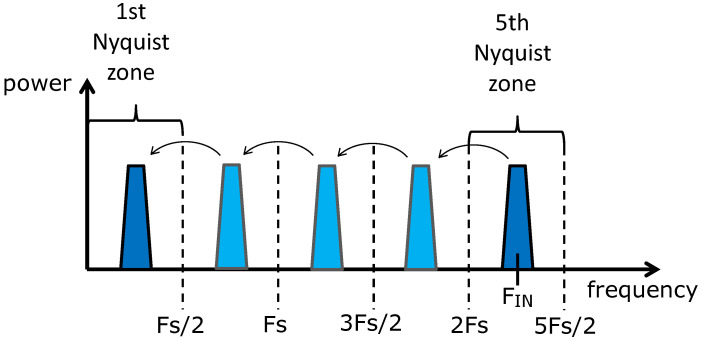
Direct sampling of an RF signal with aliasing to the 1st Nyquist zone.

Due to the developments in ADC technology, nowadays it is possible to perform direct sampling of RF signals in the GHz range without analogue downconversion, as shown in the Figure 2c. Fast (≥400 MSa/s), high-bandwidth (≥1 GHz), and high-resolution (≥14 bits) ADCs are available for this purpose [11,28]. However, in many implementations, the sampling frequency ($f_{s}$) of the ADC is substantially lower than the fundamental frequency of the modulated input signal, so that the latter must be sub-sampled in the higher Nyquist Zones (NZs) and aliased back to the first NZ to allow for acquisition, as depicted in Figure 3. In practice, sub-sampling results in a direct digital downconversion bypassing the use of an analogue mixer, hence removing its distortion effects. Moreover, fast ADCs allows for larger processing gain available in a given time-slot due to the higher $f_{s}$. However, directly sampling high input frequencies implies higher ADC noise due to clock jitter which is, in turn, subject to more demanding specifications in order to allow for the sufficient stability required by RF detection in linear accelerators.

### 3.2. IQ Sampling

Concerning digital IQ demodulation (Figure 2b,c), different sampling schemes could be applied [29]. Let us consider the following vector representation of a sinusoidal signal with frequency $f_{x}$

(1) $$\begin{array}{c} x\left(t\right)=I\sin\left(2\pi f_{x}t\right)+Q\cos\left(2\pi f_{x}t\right); \end{array}$$

where x(t) corresponds to the generic IF signal resulting from downconversion either by analogue mixing (Figure 2b), or from aliasing due to direct sampling (Figure 2c).

The most straightforward digital IQ demodulation approach is the *IQ sampling* scheme, in which the sampling frequency is chosen as:

(2) $$f_{s}=4f_{x};$$

so that x(t) is sampled exactly four times per period ($T_{x}=\frac{1}{f_{x}}$). This condition implies that the phase rotation of x(t) between two consecutive samples is equal to 90°, so that either the I or Q component can be separately selected. Indeed, the four consecutive time instants $t_{0},\ldots ,t_{3}$ result (up to an arbitrary phase offset):

(3) $$\begin{array}{c} t_{0}=0;\;t_{1}=\frac{1}{4}\frac{1}{f_{x}};\;t_{2}=\frac{2}{4}\frac{1}{f_{x}};\;t_{3}=\frac{3}{4}\frac{1}{f_{x}}; \end{array}$$

yielding the following sample sequence when applied to (1):

(4) $$\begin{array}{c} x\left(t_{0}\right)=Q;\;x\left(t_{1}\right)=I;\;x\left(t_{2}\right)=-Q;\;x\left(t_{3}\right)=-I. \end{array}$$

Then, a simple state machine can be utilized to split the ADC data stream into the IQ signals, both sampled at $\frac{f_{s}}{2}$ [30]. It should be highlighted that any ADC offset must be removed from the sampled data before demodulation, or it would result in a spurious deviation of the demodulated value [29].

Moreover, given that $f_{s}=4f_{x}$, all odd harmonics will alias exactly on the fundamental frequency $f_{x}$. This means that, in the case of non-linearities in the acquisition chain, it would not be possible to distinguish between the fundamental and odd harmonics [29], hence involving an additional spurious deviation in the measured values.

### 3.3. Non-IQ Sampling

Alternatively, *non-IQ sampling* schemes allow to estimate the IQ components with additional digital post-processing. For non-IQ sampling, the sampling frequency $f_{s}$ is taken to be an integer ratio of the input frequency $f_{x}$, such that:

(5) $$f_{s}=\frac{N}{M}f_{x},$$

where *N* and *M* are arbitrary integers. In this case, the phase rotation between consecutive samples results:

(6) $$\Delta \phi =\frac{N}{M}2\pi .$$

This choice of sampling frequency indicates that x(t) is sampled *N* times over *M* periods. The I and Q components are then estimated from the following equations, derived by means of a least squares approximation [31]:

(7) $$I=\frac{2}{N}\sum_{i=0}^{N-1}x_{i}\sin\left(i\cdot \Delta \phi \right),$$

(8) $$Q=\frac{2}{N}\sum_{i=0}^{N-1}x_{i}\cos\left(i\cdot \Delta \phi \right).$$

Equations (7) and (8) effectively represent a digital downconversion algorithm consisting of an *N*-tap moving average filter, i.e., a low-pass filter with bandwidth $\mathrm{BW}=\frac{f_{s}}{N}$. Such a solution is typically advantageous, as it intrinsically exploits coherent averaging to improve signal-to-noise ratio (SNR) by reducing the measurement noise. Conversely, given that the resonant cavities are extremely narrowband, such an averaging does not corrupt the actual signal information related to the EM field inside the cavity.

On the other hand, non-IQ algorithms introduce an additional processing delay of *N* clock periods. Nevertheless, this delay is not particularly significant at system-level, provided that the sampling frequency and clock frequency of the digital processor are sufficiently high. As compared to IQ sampling, the main advantage of non-IQ sampling is the fact that, for a chosen integer $\frac{N}{M}$ ratio, harmonics up to the (M−2)th order will fall on separate frequency bins and get, in effect, filtered out by the demodulation algorithm [32].

## 4. PolFEL LLRF System Prototype

**Figure 4:**
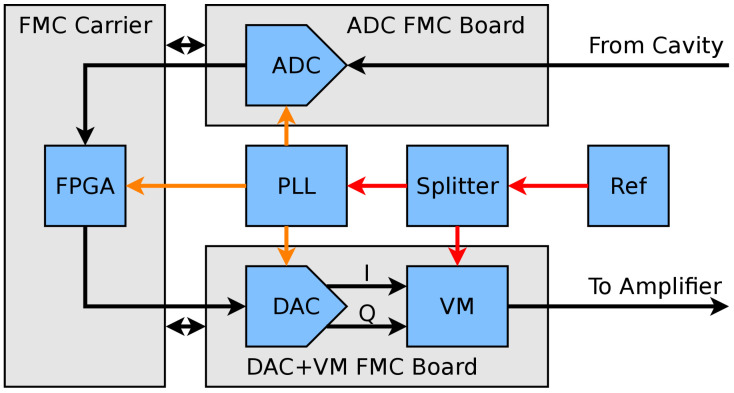
Block diagram of the prototype LLRF system for the PolFEL linear accelerator under development.

The prototype PolFEL LLRF system now under development is depicted in Figure 4. It is based on digital RF detection by direct sampling of the $f_{\mathrm{in}}$ = 1.3 GHz input signal. One LLRF system of this kind is foreseen to control a single resonant cavity with an dedicated SSPA in the final deployment of the linear accelerator.

The RF detector as well as the RF actuator are individual FPGA mezzanine cards (FMC), connected to an FPGA carrier board. This architecture provides flexibility and simplifies the servicing and upgrading of individual system components. The carrier board used in the experiments is a Xilinx KC-705 platform with a Kintex-7 FPGA chip. The RF actuator is a custom-made board equipped with a vector modulator and a dual-channel 16-bit DAC with sampling frequency of up to 500 MSa/s (AD9783 by Analog Devices). The RF detector is a commercial FMC board (ADC511 by Curtiss-Wright) including two 14-bit, 400 MSa/s ADCs (ADS5474 by Texas Instruments) featuring a wide analogue input bandwidth of up to 1.4 GHz. The chosen board is a suitable evaluation board for the target ADC. Moreover, by utilizing the FMC form factor, it offers a small size and flexibility in module replacement for future system maintenance and upgrade. In order to derive a synchronous sampling clock for the ADCs and DAC, a high performance phase-locked loop (PLL) synthesizer (LMX2582 by Texas Instruments) was used.

**Figure 5:**
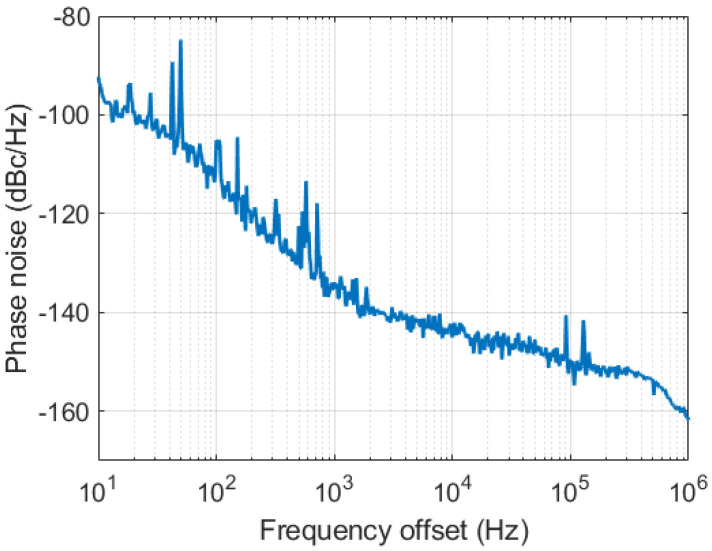
Phase noise of the 1.3 GHz reference signal generated with the Rohde and Schwarz SMA100B RF signal generator.

The phase noise characteristic of the RF source (Rohde and Schwarz SMA100B RF signal generator) used in the experimental tests as a fundamental reference was preliminarily characterized using the Agilent E5052B signal source analyzer (SSA), as shown in Figure 5. The jitter [33] of the reference signal, integrated in the 10 Hz–1 MHz band, resulted in 17.46 fs rms.

## 5. Metrological Characterization of the RF Detector

**Figure 6:**
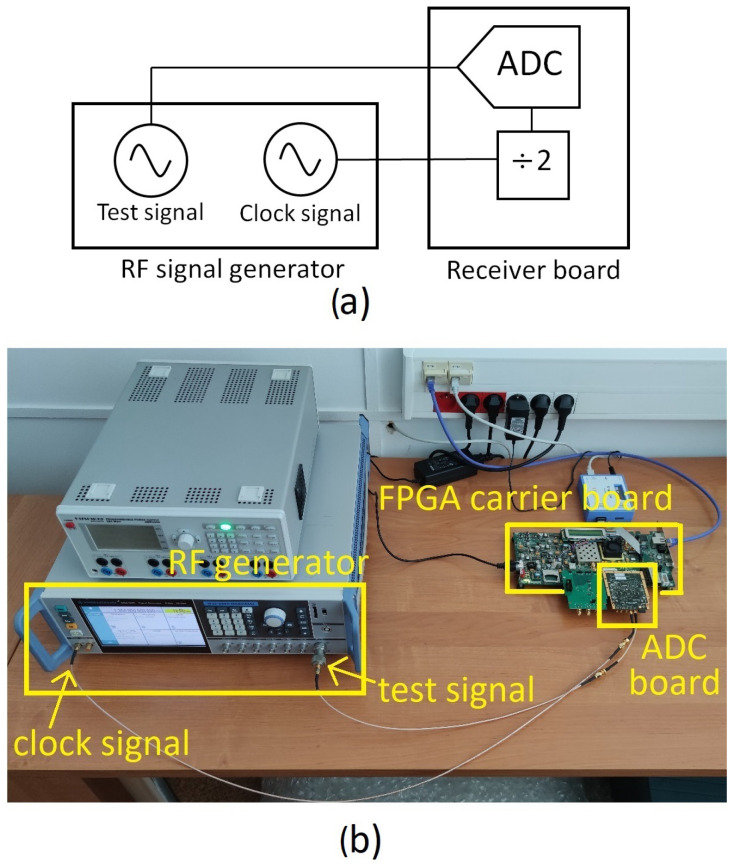
(**a**) Block diagram and (**b**) photo of the measurement set-up used for the metrological characterization of the ADC-based detector.

The broadband behavior of the ADC and its front-end circuitry are critical to the overall performance of the RF detector. Therefore, a stand-alone metrological characterization of the ADC-based detector board including the ADS5474 was firstly performed to retrieve performance metrics such as the signal-to-noise ratio (SNR), total harmonic distortion (THD), and effective number of bits (ENOB). This characterization was carried out using sine-wave excitations, in accordance with IEEE Standard 1241 [34], as similarly done in [35,36]. To this aim, a dedicated set-up was deployed, as shown in Figure 6.

In order to target the non-idealities of the ADC-based detector only, the best quality source available, i.e., the SMA100B RF signal generator, was used in the set-up of Figure 6 to generate both the sine-wave stimulus as well as a 800 MHz input clock for the ADC board. The 800 MHz input clock was characterized in terms of phase noise using the SSA, just as done for the reference signal in Section 4. The jitter of the input clock (integrated in the 10 Hz–10 MHz bandwidth) resulted in 23.66 fs rms. This input clock signal is divided by 2 on the RF detector board, resulting in an actual ADC sampling clock frequency of $f_{s}$ = 400 MSa/s.

**Figure 7:**
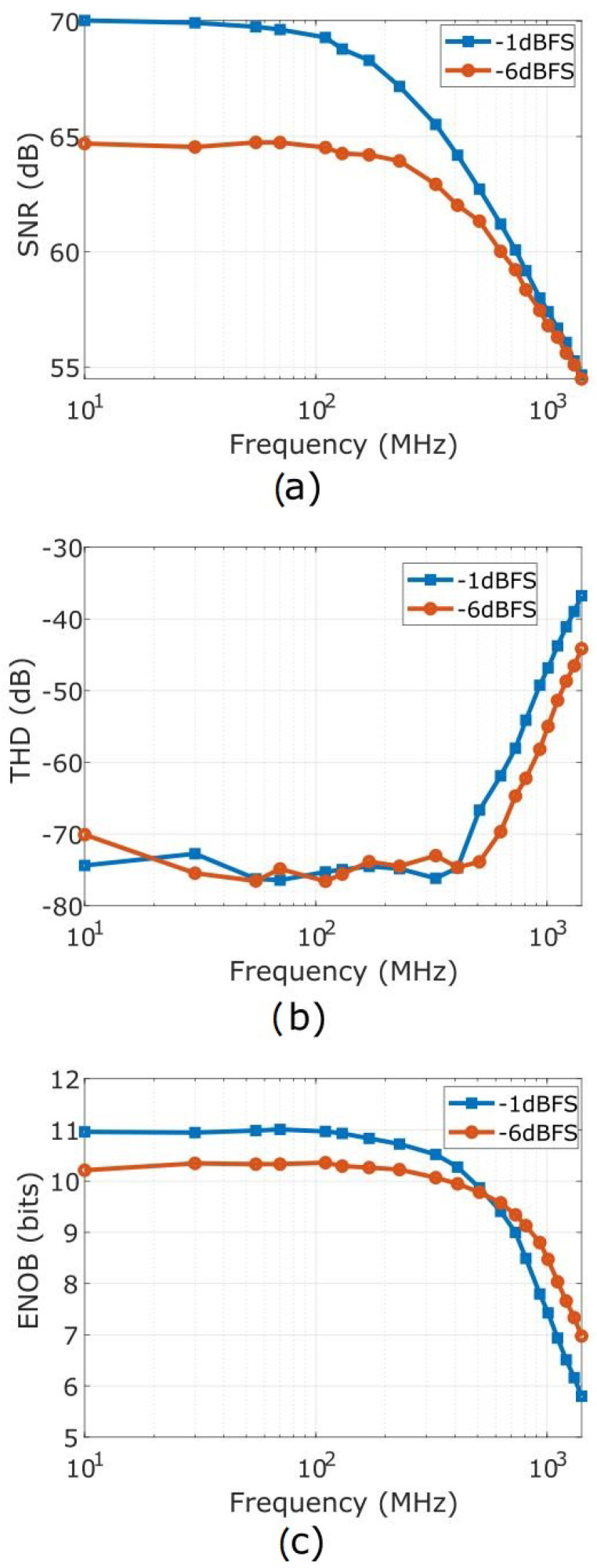
Metrological characterization of the RF detector at input amplitudes of −1 and −6 dBFS: (**a**) signal-to-noise ratio; (**b**) total harmonic distortion; (**c**) effective number of bits.

The ADC-based detector was then tested with input frequencies ranging from 10 MHz to 1.41 GHz. A record of 1 MSa was captured for each frequency at two input amplitudes, namely at −1 and −6 dBFS. The resulting SNR, THD, and ENOB across frequency are shown in Figure 7, while Table 1 presents the ADC performance metrics at $f_{\mathrm{in}}$ = 1.31 GHz, a frequency close to the final operational frequency of the target resonant cavity, yet specifically offset to avoid harmonic aliasing.

While the ENOB value at 1.31 GHz input frequency is reasonably low for this type of hardware platform, at higher frequencies it becomes mostly dominated by contributions due to harmonic distortion. Meanwhile, as discussed in Section 3, the harmonic distortion can be significantly reduced by applying the non-IQ sampling scheme and post-processing in (5)–(8). In case of IQ sampling, the even harmonics are easily filtered out, as they fall either at DC or at $\frac{f_{s}}{2}$. Therefore, the most critical parameter of the receiver for the target application is the SNR.

The SNR of the ADC-based detector is limited by thermal noise at low input frequencies, and by clock jitter at higher frequencies [37]. In general, the SNR value due to ADC noise can be calculated as follows:

(9) $${\mathrm{SNR}}_{\mathrm{ADC}}=-20\log\sqrt{{\left(10^{-\frac{{\mathrm{SNR}}_{Q}}{20}}\right)}^{2}+{\left(10^{-\frac{{\mathrm{SNR}}_{T}}{20}}\right)}^{2}+{\left(10^{-\frac{{\mathrm{SNR}}_{J}}{20}}\right)}^{2}}$$

where ${\mathrm{SNR}}_{Q}$ refers to the SNR degradation due to quantization noise, which theoretically corresponds to 86.04 dB for a 14-bit ADC. The ${\mathrm{SNR}}_{T}$, due to thermal noise, corresponds to 70.3 dB as per datasheet of the ADC [37], while ${\mathrm{SNR}}_{J}$ is due to clock jitter.

The SNR deterioration due to clock jitter depends on frequency, and it can be calculated as:

(10) $${\mathrm{SNR}}_{J}=-20\log\left(2\pi f_{\mathrm{in}}t_{J}\right),$$

where $t_{J}$ corresponds to the the rms value of the clock jitter. By fitting the SNR data visualized in Figure 7a into the formulations in (9) and (10), a total sample clock jitter $t_{J}$ of 206.35 fs is obtained. Such a comprehensive empirical value includes the external clock jitter, the aperture jitter of the ADC (corresponding to 103 fs as from datasheet [37]), as well as any spurious additive jitter.

Given that the measured value of the total sample clock jitter is substantially higher than the combination of clock source jitter (as separately measured) and aperture jitter, a significant additive jitter is likely introduced by the clock divider, and buffering circuits on the ADC board. This suggests that further minimization of the jitter, thus of the SNR, could be possibly attained by upgrading the RF detector board with a tailored design instead of the commercial solution here used.

## 6. Stability Performance of the RF Detector

### 6.1. Methodology for the Experimental Characterization

The main requirement for a LLRF system concerns the target stability of the EM field inside the resonant cavity. Stability of either the amplitude or the phase of the EM field is typically defined as the rms deviation from the corresponding mean value:

(11) $$A_{\mathrm{stab}}=\sqrt{\frac{1}{N}\sum_{i=0}^{N-1}{\left(A_{i}-\bar{A}\right)}^{2}};$$

(12) $$P_{\mathrm{stab}}=\sqrt{\frac{1}{N}\sum_{i=0}^{N-1}{\left(P_{i}-\bar{P}\right)}^{2}};$$

where $A_{\mathrm{stab}}$ and $P_{\mathrm{stab}}$ indicate amplitude and phase stability metrics, respectively, *N* is the length of the acquisition record, $A_{i}$ and $P_{i}$ are instantaneous amplitude and phase sampled values, and $\bar{A}$ and $\bar{P}$ are the mean amplitude and phase. Amplitude stability is ultimately expressed as a percentage relative to the mean amplitude value, while phase stability is expressed as an absolute deviation in degrees.

When an accelerator is operating in continuous-wave (CW) mode, any period of the excitation signal can be post-processed to retrieve the stability performance as from (11) and (12). Conversely, the stability metrics are calculated across the flat-top interval in case of pulsed-mode operation, i.e., across the time-window in which particle bunches are accelerated, and amplitude/phase of the EM field are set to be constant.

As the EM field is controlled by the LLRF feedback loop, the measurement uncertainty involved in RF detection is the main limiting factor in enforcing the target EM field stability inside the cavity. The quantification of the detector stability metrics across various receiver operating modes allows to characterize the stability performance ultimately achievable by the LLRF prototype system under development.

**Figure 8:**
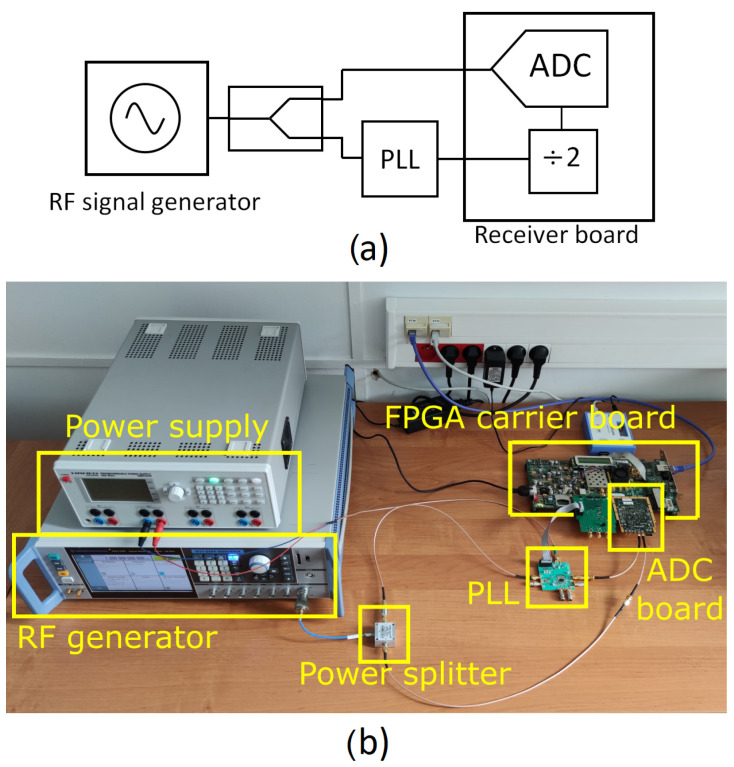
(**a**) Block diagram and (**b**) photo of the measurement set-up for the experimental evaluation of the amplitude and phase stability of the RF detector.

The measurement set-up implemented for such an assessment is shown in Figure 8. A 1.3-GHz CW RF signal is generated by the Rohde and Schwarz SMA100B, then split by a power splitter (Mini-Circuits ZFRSC-42-S+) into two channels. The first channel allows to directly feed the input of the detector board, while the second channel is used as a reference to derive a synchronous sampling clock by means of a PLL (TI LMX2582) with an integrated voltage-controlled oscillator (VCO).

**Figure 9:**
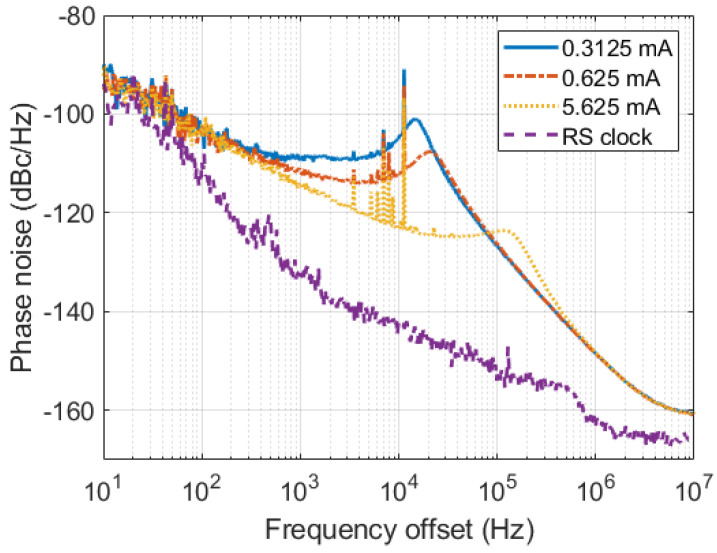
Characterization of the phase noise for an 800 MHz clock signal synthesized using LMX2582 PLL with different charge pump currents. The reference clock source by the Rohde and Schwarz SMA100B is also plotted for comparison purposes.

The loop filter bandwidth of the PLL was modified across the experiments by changing the charge-pump current in order to examine the impact of the sampling clock phase noise on the amplitude and phase stability performance of the detector. To this aim, the phase noise of the clock signal was characterized using a SSA (Agilent E5052B) for three values of the charge pump current, namely, 0.3125, 0.625 and 5.625 mA, the latter of which was found to provide optimal loop bandwidth. Figure 9 shows the phase noise performance for the 800 MHz case in the three configurations, as well as the reference clock for comparison purposes. The integrated jitter in the range from 10 Hz to 10 MHz was found to be 279.9 fs, 186.5 fs, and 95.5 fs, respectively, whereas it was 23.66 fs for the reference clock.

Another important aspect to consider when evaluating stability concerns the adopted measurement bandwidth. Under closed-loop LLRF system operation, the signal measured by the detector (including ADC noise) is compared against a set point value. A driving signal for the RF actuator is then calculated based on the deviation from the set point. The noise contribution from the detector, which is transferred to the resonant cavity via the control signal by the RF actuator, is band-pass filtered by the narrow bandwidth of the cavity, and multiplied by the gain of the feedback loop. Hence, the noise outside of the closed-loop bandwidth will have negligible impact for EM field stability, therefore it will be disregarded in this characterization. In this work, EM field stability is characterized across 10 MHz, 1 MHz, and 100 kHz measurement bandwidths realized by moving average digital filters.

### 6.2. Comparison among Sampling Schemes

When choosing a sampling scheme, we concentrated mainly on two factors, namely the sampling frequency and the number of harmonics that can be eliminated by the demodulation algorithm, as discussed in Section 3. It is advantageous to keep the sampling frequency as high as possible, in order to maintain a high level of processing gain as well as low latency when processing the data in the feedback loop.

In our case, the maximum sampling frequency for the chosen ADC is 400 MHz, which also corresponds to an IQ sampling scheme for the 1.3 GHz input signal. For comparison purposes, two other sufficiently high sampling frequencies were also considered, which represent non-IQ sampling with correspondingly different *M* and *N* values. Overall, the following sampling frequencies and demodulation configurations were used for the characterization tests of the input signal at $f_{\mathrm{in}}$ = 1.3 GHz:

- $f_{s}$ = 400 MSa/s, which corresponds to IQ sampling of the input;
- $f_{s}$ = 350 MSa/s, which corresponds to non-IQ sampling with M=5 and N=7;
- $f_{s}$ = 394.9367 MSa/s, which corresponds to non-IQ sampling with M=7 and N=24.

Since the ADC board divides the clock frequency by two, the PLL was actually programmed to synthesize frequencies of 800 MHz, 700 MHz, and 789.8734 MHz, respectively.

The stability metrics as from (11) and (12), averaged from four separate acquisitions of 131,072 samples each (corresponding to the largest record size manageable at FPGA level in this prototype), were performed for each case of sampling frequency, while the PLL charge pump current was fixed at 5.625 mA (95.5 fs jitter). Table 2 and Table 3 report the phase and amplitude stability metrics, respectively.

**Figure 10:**
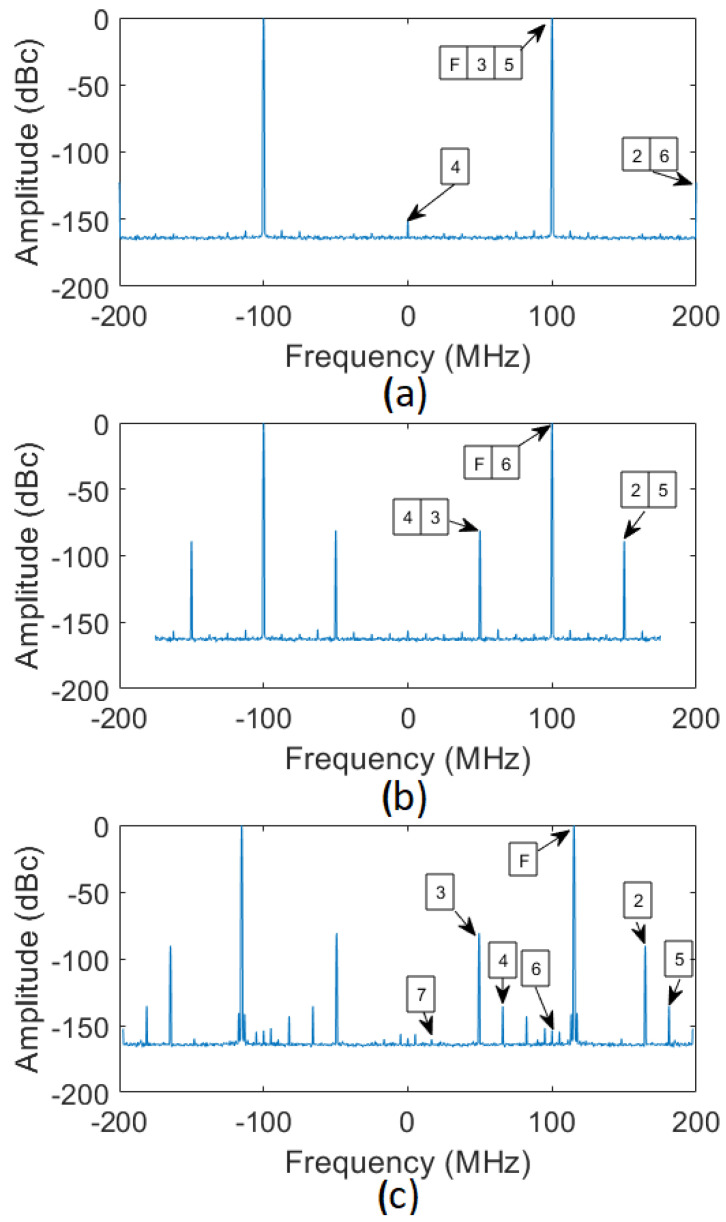
FFT of the of the acquired 1.3 GHz signal before digital demodulation (**a**) sampled at $f_{s}$ = 400 MSa/s (IQ sampling); (**b**) sampled at $f_{s}$ = 350 MSa/s (non-IQ sampling); (**c**) sampled at $f_{s}$ = 394.9367 MSa/s (non-IQ sampling). Labels indicate the harmonic order for each spectral line.

Concerning amplitude stability, the most important difference between the sampling schemes concerns the aliasing of the harmonics. As shown in Figure 10a (IQ sampling), all even harmonics are aliased only into the DC and $f_{s}$ bins, while every odd harmonic is aliased directly into the $\frac{f_{s}}{2}$ bin. This makes odd harmonics indistinguishable from the input CW signal and introduces an unknown deviation into the measurement. On the other hand, in Figure 10b,c (non-IQ sampling), all harmonics up to the 5th and 22nd order, respectively, fall away from the the demodulated input frequency, and are inherently filtered out by the demodulation algorithm.

Overall, for the reported measurement bandwidths, all three cases display similar values for amplitude stability, and all three sampling frequencies enable sufficient amplitude measurement accuracy. Nevertheless, for the IQ sampling case, any amplitude instability due to higher harmonic are superimposed in frequency to the input tone due to aliasing. Hence, it cannot be further reduced by using an increasingly narrower measurement bandwidth. Conversely, a narrower measurement bandwidth enables proportionally higher stability in case of non-IQ sampling.

All three cases display similar values for phase stability. Indeed, as will be shown in Section 6.3, phase stability is largely determined by the clock signal jitter when using direct sampling whereas, in this test, the three clock frequencies are generated using the same PLL loop bandwidth and RF input signal.

### 6.3. Impact of Clock Signal Phase Noise

The amplitude and phase stability have been measured for an IQ sampling scheme with $f_{s}$ = 400 MSa/s and different clock source configurations (as previously shown in Figure 9) in order to examine the impact of clock phase noise. As previously done, four acquisitions of 131,072 points are averaged for obtaining the stability metrics. Table 4 and Table 5 report the measured amplitude and phase stability, respectively, for all test cases. A clear impact of the clock jitter can be noticed, especially concerning phase stability.

**Figure 11:**
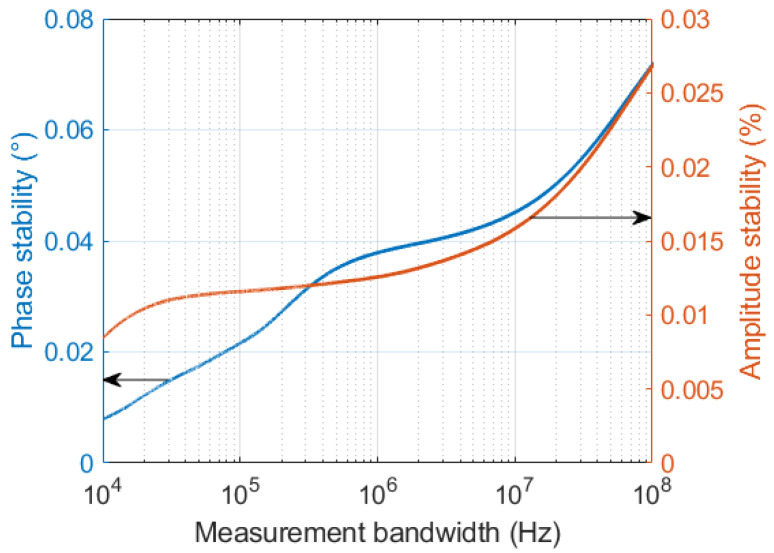
Measured phase and amplitude stability across different measurement bandwidths.

Figure 11 shows the dependency of the stability metrics against the measurement bandwidth in the case of PLL charge-pump current of 5.625 mA, which corresponds to the lowest jitter. In particular, the plot shows the highest rate of change of the phase stability metric at measurement bandwidths narrower than 1 MHz. This behaviour can be interpreted considering that the phase noise of the sampling clock is convoluted with the input tone in the sampling process, whereas wideband noise in the acquisition is uniformly distributed across frequency, so that the former dominates the stability behavior in the narrow bandwidth around the demodulated input tone.

This aspect can be further analyzed by converting the measured rms phase stability to RMS time deviation by means of the following conversion $t_{\mathrm{stab}}=\frac{P_{\mathrm{stab}}}{360^{{}^{\circ}}f_{\mathrm{in}}}$, where $P_{\mathrm{stab}}$ is the rms phase stability in degrees, as defined in (12), and $f_{\mathrm{in}}$ is the input frequency. The rms time deviations ($t_{\mathrm{stab}}$) can then be directly compared against the rms time jitter ($t_{J}$) of the sampling clock signal. For this purpose, the phase noise of the sampling clock in the case of a PLL charge pump current of 5.625 mA was integrated in different frequency bandwidths. The lower limit of the integration bandwidth was set as $f_{\mathrm{low}}=\frac{f_{s}}{2N}$, where $f_{s}$ is the sampling frequency and *N* is the length of the record. Indeed, frequencies smaller than $f_{\mathrm{low}}$ do not impact the measurement due to the finite record length [38]. The upper limit of the integration bandwidth $f_{\mathrm{high}}$ was set equal to half of the desired measurement bandwidth, in order to account for both sidebands of the phase noise spectrum.

**Figure 12:**
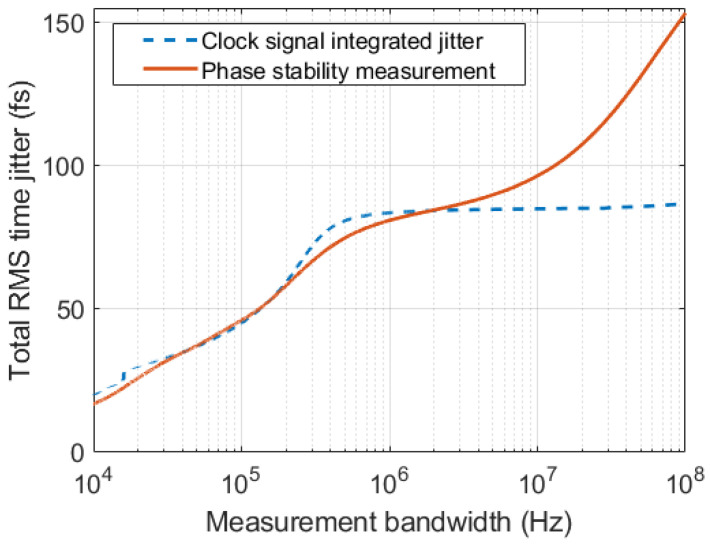
Comparison of rms time deviation calculated from the measured phase stability against the integrated sampling clock jitter.

Figure 12 shows the comparison of the integrated clock jitter ($t_{J}$) against the jitter calculated from phase stability measurement ($t_{\mathrm{stab}}$) across the same set of measurement bandwidths as in Figure 11. The quantities are approximately aligned for measurement bandwidths up to around 1 MHz, confirming that the noise behavior at narrow measurement bandwidths is mainly due to the jitter of the clock. Beyond that value, the noise in the acquisition is instead dominated by the noise floor of the ADC.

**Figure 13:**
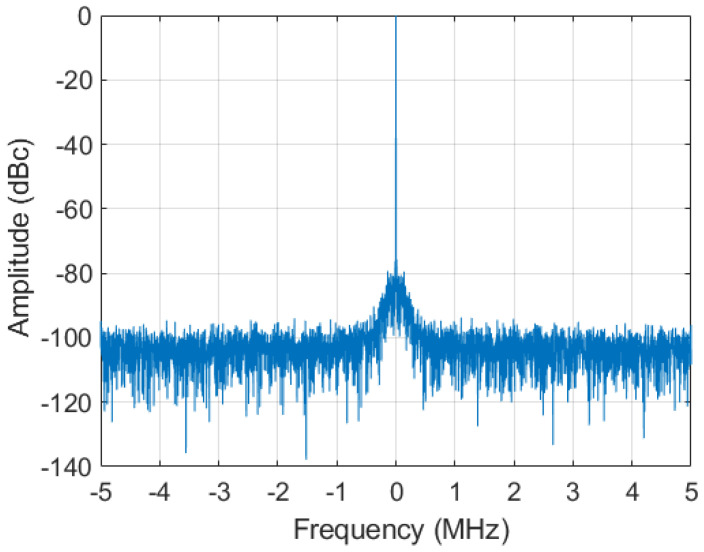
FFT of the demodulated 1.3-GHz input signal in the −5 MHz to 5 MHz range.

This can also be observed by evaluating the 131,072-point FFT of the demodulated signal in Figure 13. The spectrum is shown in a range from −5 MHz to 5 MHz, and the typical power spectral density (PSD) profile of the phase noise can be noticed in a bandwidth from −0.5 to 0.5 MHz around the demodulated input tone. These results demonstrate the dominant impact of the clock signal phase noise on the achievable phase stability in an LLRF system.

## 7. Conclusions

The deployment and performance evaluation of a prototype RF detector within the LLRF system of the PolFEL linear accelerator have been described in the article. The prototype embeds a 400 MSa/s ADC featuring a wide analogue bandwidth, which allows for the direct sampling and digital demodulation of the 1.3 GHz input signal of the resonant cavity by employing sub-sampling schemes.

The ADC receiver was first evaluated in terms of its fundamental parameters, yielding SNR of a ∼55 dB and THD of ∼39 dB at the target frequency of application. These results justify the use of the considered ADC configuration for implementing signal acquisition by direct sampling.

The detector was then evaluated in terms of achievable amplitude and phase stability within the LLRF system. By using a high-performance PLL circuit with an integrated VCO for generating the clock signal (as employed in the LLRF system prototype), amplitude and phase stability tests were performed for three different sampling frequencies, corresponding to different sub-sampling schemes, as well as for controlled phase noise values. While amplitude stability in the order of 0.01% could be easily achieved by means of the high processing gain exploiting the fast-sampling ADC, phase stability resulted to be more sensitive to clock signal characteristics.

Phase stability from ∼0.04° to ∼0.02° were measured, depending on the adopted measurement bandwidth. Nevertheless, phase stability below 0.01° could be achieved with a higher quality clock source. These experimental results allowed to demonstrate that the performance of the RF detector utilizing direct sampling is largely limited by the the clock signal phase noise.

Depending on the ultimate requirements for the PolFEL linear accelerator, the procedures and tools implemented in this work will allow for the evaluation of the stability performance of the finalized system. Overall, the experimental results here reported are expected be in line with the specifications, leading to a very simplified LLRF system architecture, with minimized latency and no additional hardware for LO clock signal generation and downconversion. Future work will investigate the proposed detection as well as suitable digital filtering in the presence of other cavity passband modes.

## Figures and Tables

**Table 1:** Measured detector parameters at $f_{\mathrm{in}}$ = 1.31 GHz.

Amplitude	SNR (dB)	THD (dB)	ENOB
−1 dBFS	55.26	−38.95	6.16
−6 dBFS	55.10	−46.53	7.33

**Table 2:** Rms amplitude stability measured across different bandwidths for different sampling schemes and frequencies.

Sampling Scheme	Sampling Frequency (MSa/s)	Measurement Bandwidth		
		10 MHz	1 MHz	100 kHz
IQ	400	0.016%	0.013%	0.011%
non-IQ	350	0.026%	0.011%	0.006%
non-IQ	394.9367	0.021%	0.009%	0.005%

**Table 3:** Rms phase stability measured across different bandwidths for different sampling schemes and frequencies.

Sampling Scheme	Sampling Frequency (MSa/s)	Measurement Bandwidth		
		10 MHz	1 MHz	100 kHz
IQ	400	0.045°	0.038°	0.021°
non-IQ	350	0.044°	0.036°	0.021°
non-IQ	394.9367	0.049°	0.039°	0.023°

**Table 4:** Rms amplitude stability measured in different bandwidths for different charge-pump currents of the clock PLL, compared to the reference clock source (Rohde & Schwarz SMA100B).

PLL Charge Pump Current	Jitter (fs)	Measurement Bandwidth		
		10 MHz	1 MHz	100 kHz
0.3125 mA	279.9	0.023%	0.014%	0.012%
0.625 mA	186.5	0.024%	0.013%	0.011%
5.625 mA	95.5	0.016%	0.013%	0.012%
Ref clock	23.66	0.010%	0.005%	0.002%

**Table 5:** Rms phase stability measured in different bandwidths for different charge pump current of the clock PLL, compared to the Rohde and Schwarz SMA100B clock source.

PLL Charge Pump Current	Jitter (fs)	Measurement Bandwidth		
		10 MHz	1 MHz	100 kHz
0.3125 mA	279.9	0.131°	0.130°	0.121°
0.625 mA	186.5	0.082°	0.080°	0.070°
5.625 mA	95.5	0.045°	0.038°	0.021°
Ref clock	23.66	0.033°	0.014°	0.007°
